# Eco-friendly four spectrophotometric approaches for the simultaneous determination of the recently FDA-approved combination, bupivacaine and meloxicam in pharmaceutical dosage forms

**DOI:** 10.1038/s41598-024-77562-9

**Published:** 2024-11-11

**Authors:** Aya Saad Radwan, Mohamed M. Salim, Fathalla Belal, Galal Magdy

**Affiliations:** 1 Department of Pharmaceutical Chemistry, Faculty of Pharmacy, Horus University-Egypt, New Damietta, Egypt; 2 Pharmaceutical Analytical Chemistry Department, Faculty of Pharmacy, Mansoura University, Mansoura, 35516 Egypt; 3 Pharmaceutical Analytical Chemistry Department, Faculty of Pharmacy, Kafrelsheikh University, Kafrelsheikh, 33511 Egypt; 4 Department of Pharmaceutical Analytical Chemistry, Faculty of Pharmacy, Mansoura National University, Gamasa, 7731168 Egypt

**Keywords:** Bupivacaine, Meloxicam, Spectrophotometry, Greenness, Chemistry, Analytical chemistry

## Abstract

In this study, bupivacaine (BUP) and meloxicam (MLX) were simultaneously assayed in their co-formulated ampoules without interference using four affordable, sensitive, and eco-friendly spectrophotometric methods. The assay of MLX at 359.3 nm over the concentration range of 1.0–15.0 µg/mL was accomplished using a direct UV-spectrophotometric method (Method I) without interference from BUP. However, there was a significant overlap between the spectra of BUP and MLX, making it difficult to determine BUP directly from the UV spectrum. Therefore, various UV-based techniques, including second derivative spectrophotometry (Method II), ratio subtraction method (Method III), and absorption factor method (Method IV), were used to determine BUP over the concentration range of 5.0–80.0 µg/mL. The proposed methods could simultaneously determine the studied drugs with a challenging ratio of 33.3:1.0 (BUP: MLX), which increases the importance of the current study. The proposed methods were applied to estimate the studied drugs in commercial ampoules with high % recoveries and low %RSD values. The excellent eco-friendliness of the developed methods was demonstrated using GAPI and AGREE metrics. The developed methods were validated according to ICHQ2(R2) guidelines. The proposed methods can be better suited for the routine analysis of BUP and MLX in their fixed-dose combination with high selectivity.

## Introduction

**Fig. 1:**
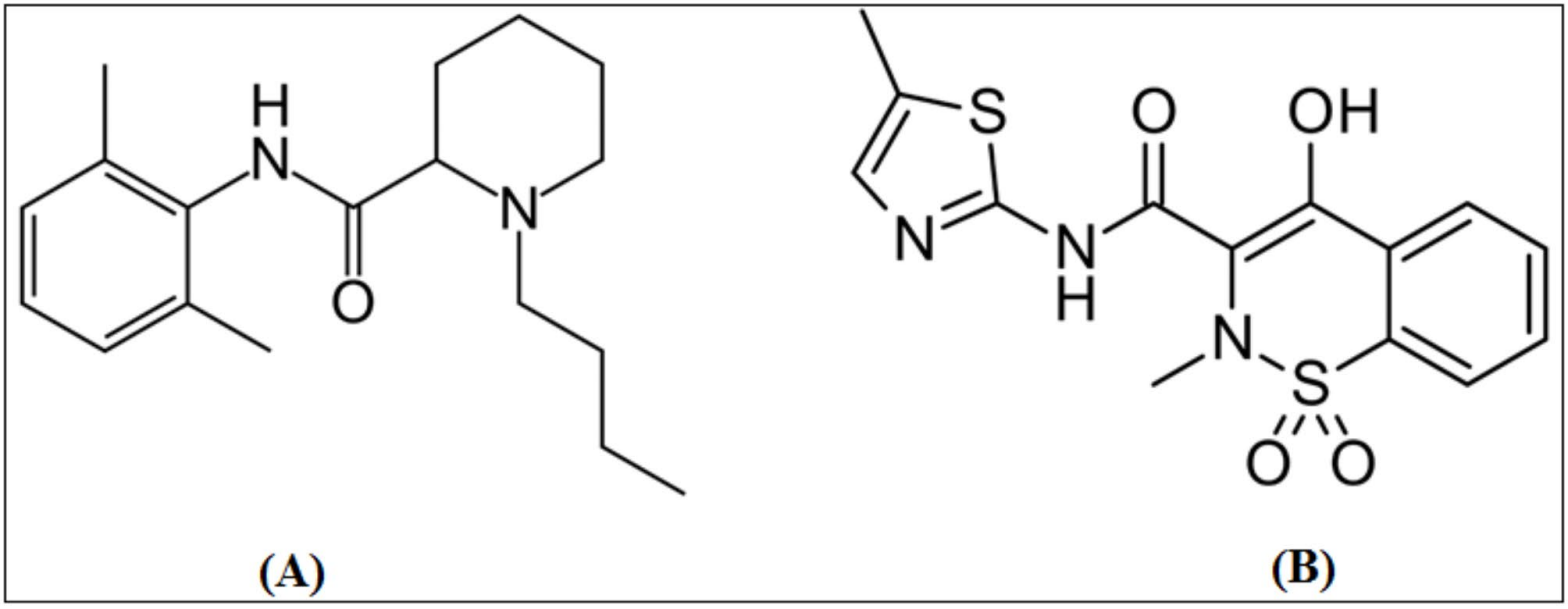
Chemical formulae of Bupivacaine (**A**) and Meloxicam (**B**).

Bupivacaine (BUP) is a long-acting amide anaesthetic. Chemically, it is (RS)-1-butyl-N-(2,6-dimethyl phenyl) piperidine-2-carboxamide (Fig. 1A). In high doses, it works by blocking the nerve impulses that flow from sensory nerve terminals to motor nerve endings^1^.

Meloxicam (MLX), is a non-steroidal anti-inflammatory medication (NSAID) from the enolic acid family that has been found to inhibit COX-2. Chemically, it is described as 4-hydroxy-2-methyl-(5-methyl-2-thiazolyl)-2H-1,2-benzothiazine-3-carboxamide-1,1-dioxide (Fig. 1B). MLX is used to treat osteoarthritis, rheumatoid arthritis, and other joint conditions^2^. Bupivacaine/meloxicam is a fixed-dose mixture of the NSAID meloxicam and the local anaesthetic bupivacaine, which is frequently indicated to relieve postoperative pain in the EU and USA^3^. For the determination of BUP, a variety of analytical techniques were reported, including spectrophotometry^4^, chromatography^5–9^, and electrochemistry^10^, while MLX has been assayed through the use of different spectroscopic methods^11–15^. Furthermore, MLX analysis in various dosage forms and biological matrices was reported in various chromatographic methods^16–19^.

Meanwhile, for the concurrent assay of BUP and MLX in co-formulated dosage forms, only two chromatographic methods^20,21^ have been published. In addition, two spectrophotometric methods were also recently reported for their concurrent analysis^22,23^. When compared to the reported chromatographic methods, the developed spectrophotometric methods are more affordable and straightforward, require less expensive instrumentation and small volumes of organic solvents, don’t need prior separation steps, and are more eco-friendly^24^. In addition, the proposed methods could simultaneously determine the studied drugs with a challenging ratio of 33.3:1.0 (BUP: MLX), magnifying the current study’s importance. Compared to the recently reported spectrophotometric methods, the developed methods in this work applied different UV-based techniques (second derivative spectrophotometry (Method II), ratio subtraction method (Method III), and absorption factor method (Method IV)). They exhibited about 20-fold higher sensitivity than the spectrophotometric method reported by El-Malla et al.^22^. Moreover, the developed methods showed tenfold higher sensitivity for BUP than the method reported by Bahgat et al.^23^. Therefore, the established procedures can be better suited for the routine analysis of BUP and MLX with high sensitivity and selectivity in medicinal formulations that combine both substances. Moreover, the green profile and ecofriendliness of the developed approaches were successfully evaluated using the Green Analytical Procedure Index (GAPI) and Analytical GREENNESS metric approach (AGREE), proving the excellent greenness of the proposed methods^25,26^.

## Experimental

### Apparatus

Shimadzu UV–Visible double-beam spectrophotometer (Kyoto, Japan) was utilized to conduct the spectrophotometric analyses. It was equipped with matching quartz cuvettes with a 1 cm path length. The 200–600 nm wavelength region of the absorption spectra of both medications was recorded using auto-sampling intervals, 1 nm slit width, and a rapid scan speed.

Using ∆λ = 4 nm and a scale factor of 100, the second derivative spectra for BUP were obtained.

### Materials and reagents

HPLC-grade ethanol, acetonitrile, and methanol were obtained from Fisher Scientific UK, Loughborough, Leicestershire, UK. NaOH and HCl were provided by El-Nasr Pharmaceutical Co., Cairo, Egypt.

Bupivacaine HCl (99.90% purity) was generously supplied by Hikma Pharmaceuticals, 6th of October City, Giza, Egypt.

Meloxicam (99.40% purity) was kindly supplied by ADWIA, Cairo, Egypt.

### Stock standard solutions

1000.0 µg/mL BUP and MLX stock solutions were prepared by dissolving 25.0 mg of each compound in methanol in a 25.0 mL volumetric flask. When maintained in a refrigerator at 2 °C, the solutions were shown to be stable for at least two weeks.

### Calibration curves

Zero-order absorption spectra were measured with respect to methanol, which served as a reference. Accurately measured volumes of the stock solutions were transferred into a set of measuring flasks (10.0 mL), diluted to the desired concentration with the same solvent, and thoroughly mixed to get the final concentration in the range of (5.0–80.0) µg/mL of BUP and (1.0–15.0) µg/mL of MLX. The final drug concentrations (µg/mL) were plotted versus the absorbance values to create the calibration graphs, and the relevant regression equations were then derived.

#### Method (I): direct UV

Without interference from BUP, MLX was determined at λ 359.3 nm, as described under Section "Calibration curves".

#### Method (II): second derivative (D^2^)

The D^2^ amplitude values for BUP were recorded at 245.1 nm. The calibration graph and accompanying regression equation were then obtained by plotting the derivative amplitudes against the corresponding concentration.

#### Method (III): ratio subtraction (RS)

**Fig. 2:**
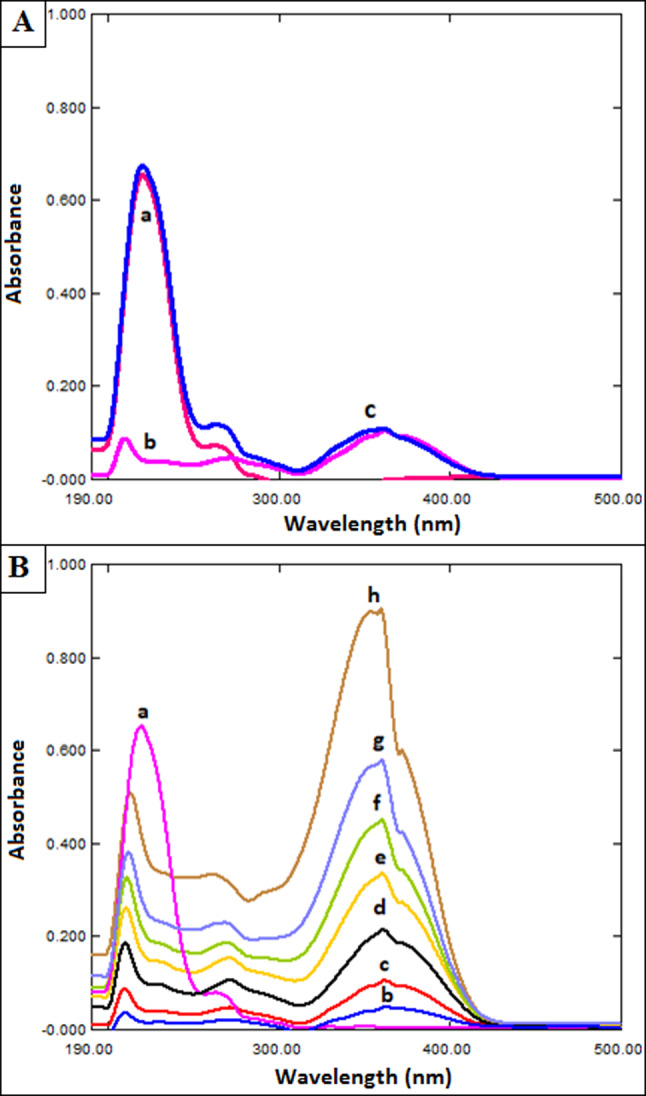
(**A**) Zero order absorption spectra of BUP (a; 33.3 μg/mL), MLX (b; 1.0 μg/mL), and mixture (c) in methanol, (**B**) Direct UV spectrophotometric spectra of MLX (b-h: 1.0, 2.0, 4.0, 6.0, 8.0, 10.0, 15.0 μg/mL) and a fixed concentration of BUP (a) (60.0 μg/mL).

The RS method was used to detach the MLX spectrum for BUP estimation. The two drugs, BUP (A) and MLX (B), have overlapping spectra, with MLX’s spectrum being longer than that of BUP beyond 300 nm (Fig. 2). By dividing the overlapping spectra of the synthetic mixtures (A + B) by a carefully chosen MLX concentration (4.0 µg/mL) of standard B’ (divisor), an innovative ratio spectrum ([A + B] / B') is created. As BUP (A) has no absorbance in the plateau region (300–500 nm), the division obtained constant values of absorbance that is equal to (B / B') along this region. The ratio spectrum ([A + B] / B') is subtracted from the fixed absorbance values (B / B') to produce a new (A / B') spectrum. The original spectrum (A) was then produced by multiplying the acquired (A / B') spectrum by (B'). This obtained spectrum was then utilized to acquire the appropriate regression equation and determine BUP (A) at 224.7 nm.

#### Method (IV): absorption factor (AF) method

The first step was to create a calibration curve for BUP by graphing different concentrations (ranging from 5.0 to 80.0 µg/mL) against zero order absorbance amplitudes (A) at 224.7 nm (λ_1_). Second, the calibration curve for MLX was obtained by graphing different concentrations (ranging from 1.0 to 15.0 µg/mL) against zero-order absorbance amplitudes (A) at 224.7 nm (λ_1_). Thirdly, the zero-order absorbance amplitudes (A) at 359.3 nm (λ_2_) (λ_max_) against various concentrations (ranging from 1.0 to 15.0 µg/mL) were graphed to create the calibration curve for the MLX.

### Laboratory-prepared mixtures

Because ampoules containing BUP and MLX are not available in Egyptian retailers, the laboratory-prepared mixture was used to mimic the dosage form. In a 100.0 mL measuring flask, methanol was used to dissolve precisely weighed amounts of 29.25 mg BUP, and 0.88 mg MLX with triacetin, dimethyl sulfoxide, maleic acid, and triethylene glycol/triethylene glycol polyglycolide copolymer as excipients. Then the procedures outlined under Section "Calibration curves" were used.

## Results and discussion

As shown in Fig. 2, the overlay of the UV-spectra of BUP and MLX (in the ratio 33.3:1.0, BUP: MLX) demonstrates that MLX may be identified at 359.3 nm without interference from BUP, the regression equation was constructed for MLX at λ 359.3 nm as summarized in Table 1.

**Table 1:** Analytical performance data for the estimation of BUP and MLX by the developed approaches.

Parameter	BUP			MLX	
Methods	D^2^	RS	λ_max_	Direct	λ_max_
Wavelength (nm)	245.1	224.7	224.7	359.3	224.7
Linearity range (µg/mL)	5.0–80.0	5.0–80.0	1.0–25.0	1.0–15.0	0.6–10.0
Intercept (a)	-1.9 × 10^–3^	6.5 × 10^–2^	7.7 × 10^–2^	-2.9 × 10^–2^	-1.5 × 10^–3^
Slope (b)	4.1 × 10^–3^	8.7 × 10^–3^	9.1 × 10^–3^	6.1 × 10^–2^	2.4 × 10^–2^
Correlation coefficient (r)	0.9999	0.9998	0.9999	0.9999	0.9998
S.D. of residuals (Sy/x)	1.5 × 10^–2^	6.0 × 10^–3^	1.2 × 10^–3^	4.8 × 10^–3^	2.4 × 10^–3^
S.D. of intercept (Sa)	1.9 × 10^–4^	7.7 × 10^–4^	1.5 × 10^–4^	6.9 × 10^–4^	3.4 × 10^–4^
S.D. of slope (Sb)	2.0 × 10^–5^	9.0 × 10^–5^	2.0 × 10^–5^	4.0 × 10^–4^	2.0 × 10^–4^
% RSD	0.762	1.170	0.730	1.132	1.392
% Error	0.288	0.444	0.275	0.430	0.530
LOD (µg/mL)	0.16	0.29	0.05	0.04	0.05
LOQ (µg/mL)	0.47	0.88	0.16	0.11	0.15

However, there was a significant overlap between the spectra of BUP and MLX, making it difficult to identify BUP from a straight UV-spectrum. Such a challenge was handled by manipulation of UV-spectra utilizing multiple multi-component UV spectrophotometric approaches.

In order to simultaneously analyze BUP and MLX in a challenging ratio of 33.3: 1.0, four straightforward, quick, accurate, and green spectrophotometric methods are developed in this work. These methods included D^2^, RS, AF, and direct UV procedures (for BUP).

### Method optimization

The impact of various variables on the performance of the suggested methods was extensively examined.

#### Selection of solvent

For the study of BUP and MLX, several solvents with different polarities, including distilled water, ethanol, methanol, acetonitrile, and aqueous solvents with various pH values, such as 0.1 M NaOH, 0.1 M HCl, borate buffer of pH 8, and acetate buffer of pH 4 were investigated. In order to enable the quantification of MLX in its combination with BUP, it was critical to improve MLX absorbance. After several trials, methanol demonstrated improved solubility properties and absorption intensities for both drugs. Therefore, to make the established procedures more sensitive, methanol was chosen for the preparation of the BUP and MLX solutions.

#### Scaling factor and delta lambda

Different scaling factor (SF) and delta lambda (∆λ) values were investigated in order to improve 2D performance. The best values were found to be ∆λ = 4 nm and SF = 100 after smoothing at ∆λ = 8 nm and SF = 1, which offered the highest absorption intensity with the least amount of noise, particularly in the case of low concentrations.

### Methods’ characteristics

#### Method I: direct UV approach for MLX

As shown in Fig. 2, MLX can be measured at 359.3 nm without BUP interference.

#### Method II: ^2^D spectrophotometric approach for determination of BUP

**Fig. 3:**
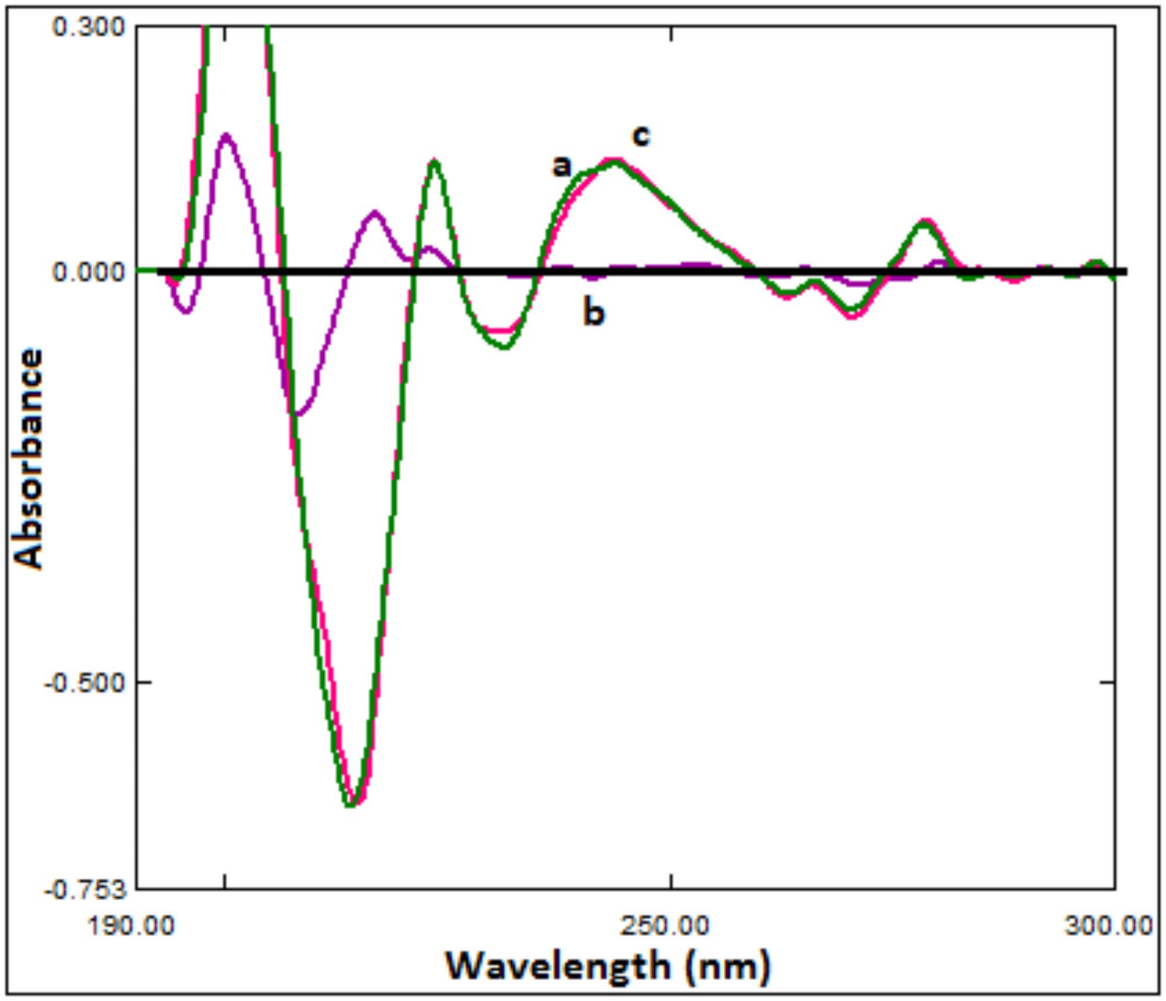
Second derivative spectra of BUP (**a** 33.3 μg/mL), MLX (**b** 1.0 μg/mL) and a mixture of BUP (33.3 μg/mL) and MLX (1.0 μg/mL) (**c**) in methanol.

BUP was quantified by derivative spectrophotometry with zero-crossing points. As demonstrated in Fig. 3, using ∆λ = 4 nm and SF = 100, the zero-order UV spectrum of the binary mixture was converted into 2D. The wavelength used for BUP measurements was 245.1 nm since it displayed the highest 2D amplitude results (without MLX interference). Table 1 provides an illustration of the statistical characteristics of calibration curves.

#### Method III: ratio subtraction (RS) approach for BUP

**Fig. 4:**
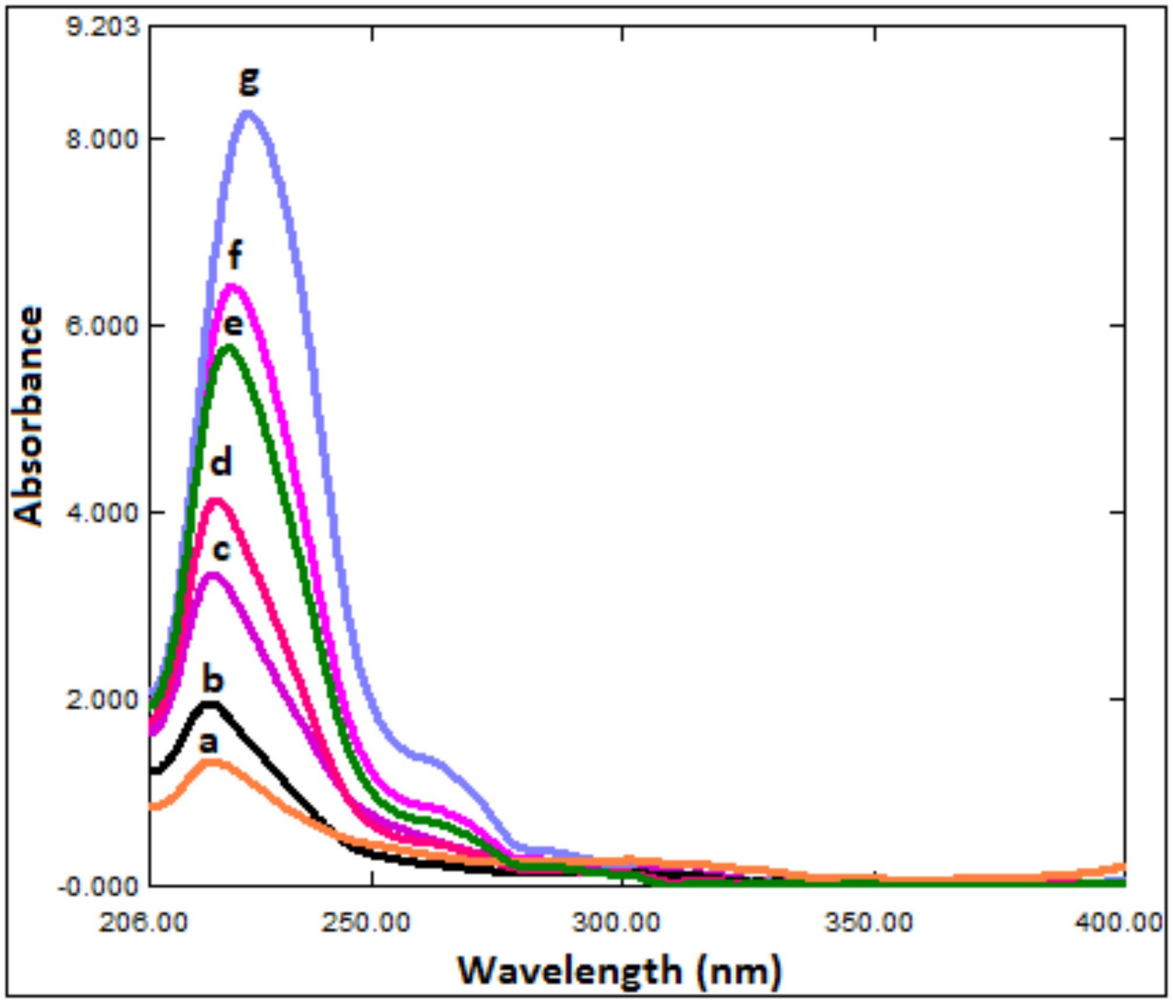
Ratio spectrophotometric spectra of different concentrations of BUP using 3.0 μg/mL MLX as a divisor, where: (**a**-**g**) BUP at concentrations of (1.0, 2.0, 6.0, 10.0, 16.0, 20.0, 25.0 μg/mL).

**Fig. 5:**
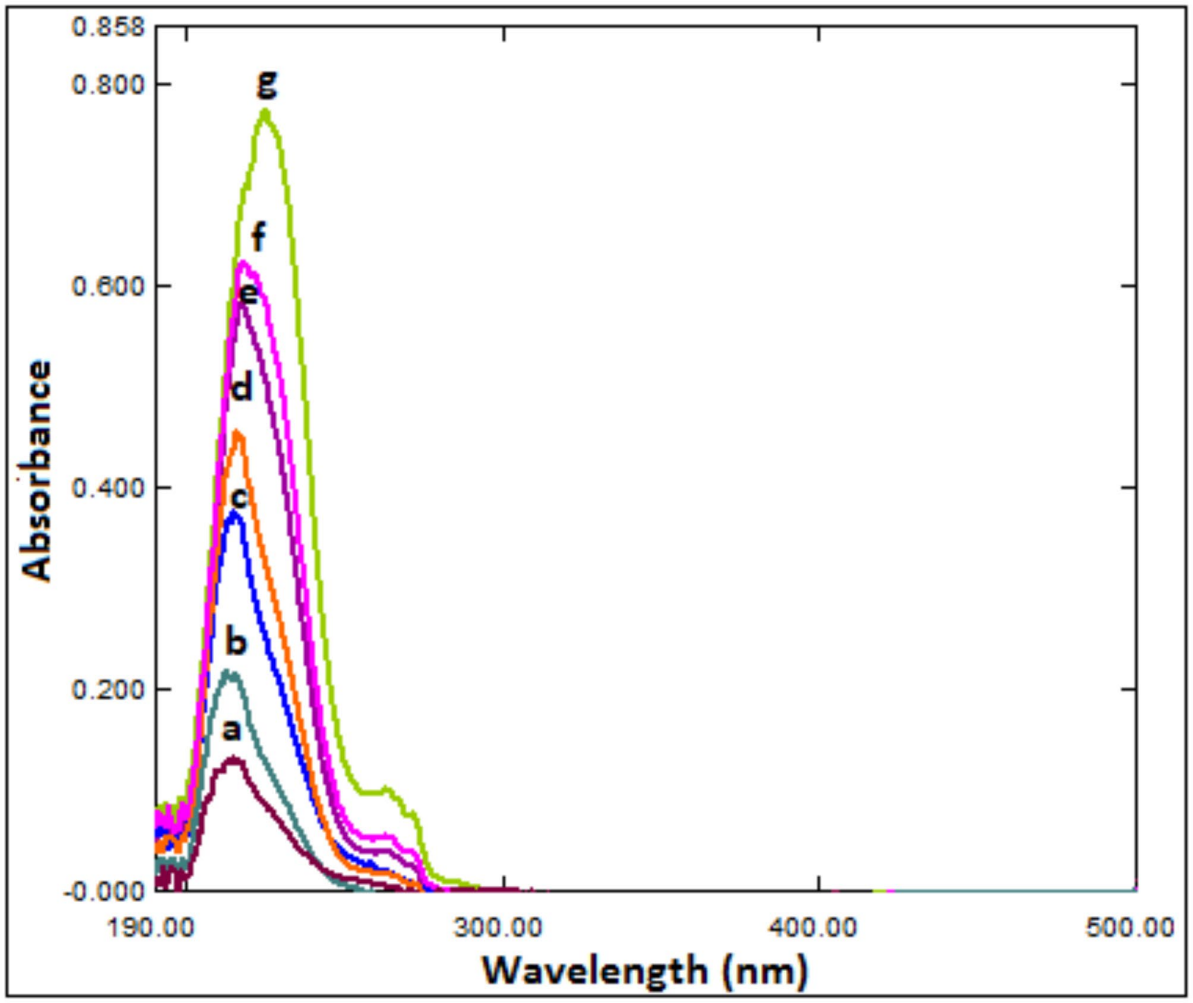
Ratio subtraction UV spectrophotometric spectra of BUP using MLX as a divisor (3.0 μg/mL), where: (**a**-**g**) BUP at concentrations of (1.0, 2.0, 6.0, 10.0, 16.0, 20.0, 25.0 μg/mL).

RS was used to remove the MLX spectrum. The method is based on the finding that the concentration of (A) can be calculated by dividing the zero-order absorption spectra of the laboratory-prepared mixtures (BUP and MLX) by (4.0 µg/mL) of standard MLX (B' = divisor) for a mixture of two drugs, BUP (A) and MLX (B), whose spectra overlap and (B) spectrum is extended beyond than that of (A) (Fig. 2). The new ratio spectrum presented in Fig. 4 is produced by multiplying the obtained spectra by the divisor (B'). The produced spectra are then multiplied by (B'), the divisor, after subtracting the absorbance values of these constants (B/B') in the plateau region (307–500 nm). A direct determination of BUP at 224.7 nm was then made using the original spectrum of (A) (Fig. 5).

#### Method IV: AF approach for BUP

At 359.3 nm (λ_max_ of MLX), the concentration was calculated from its regression equation, and at 224.7 nm (λ_max_ of BUP), the concentration was calculated by subtracting the absorption caused by the interfering drug.

The following equations were used to quantitatively estimate the amounts of BUP and MLX in the mixture:

1) Concentration of MLX at 359.3 nm

1 $${\text{C}}_{{{\text{MLX}}}} \left( {\mu {\text{g}}/{\text{mL}}} \right) = \left[ {{\text{A}}_{{{\text{MLX}}}} + {\text{ intercept }}\left( {0.0{293}} \right)} \right]/{\text{slope}}\;\left( {0.0{6}0{8}} \right)\quad {\text{r }} = 0.{9999}$$

2) Calculated absorbance of MLX at 224.7 nm

2 $${\text{A}}_{{{\text{MLX}}}} \left( {{\text{calculated}}} \right) = 0.0{\text{236 C}}_{{{\text{MLX}}}} - \, 0.00{15} \quad {\text{r }} = 0.{9998}$$

3) So, the corrected absorbance of BUP at 224.7 nm

3 $${\text{A}}_{{{\text{BUP}}}} \left( {{\text{corrected}}} \right) = {\text{A}}_{{{\text{mix}}}} {-}{\text{A}}_{{{\text{MLX}}}} \left( {{\text{calculated}}} \right) = 0.00{\text{91 C}}_{{{\text{BUP}}}} + \, 0.0{769} \quad {\text{r}} = 0.{9999}$$

4) Calculation of BUP concentration at 224.7 nm

4 $${\text{C}}_{{{\text{BUP}}}} \left( {\mu {\text{g}}/{\text{mL}}} \right) = \left[ {{\text{A}}_{{{\text{BUP}}}} \left( {{\text{corrected}}} \right){-}{\text{intercept}}\left( {0.0{769}} \right)} \right]/{\text{slope}}\left( {0.00{91}} \right)\quad {\text{r}} = 0.{9999}$$

### Methods’ validation

According to ICHQ2 (R2) guidelines^27^, the precision, accuracy, linearity, range, limit of quantitation (LOQ), limit of detection (LOD), and selectivity of the proposed methods were all estimated.

#### Linearity and range

Seven concentrations of BUP and MLX in the ranges of (5.0–80.0) and (1.0–15.0) µg/mL, respectively, were used to create calibration plots. Both BUP and MLX revealed linear relationships between concentrations and the obtained response. High values of the correlation coefficients (r) and low values of the standard deviation of intercepts (S_a_), the standard deviation of residuals (S_y/x_), and the standard deviation of slopes (S_b_) were used to validate good linearity of the developed methods, as presented in Table 1.

#### LOD and LOQ

The following equations were used to establish the LOD and LOQ for BUP and MLX according to ICHQ2 (R2) guidelines.

$${\text{LOD}} = 3.3\;S{\text{a}}/{\text{b}}$$

$${\text{LOQ}} = {10}\;{\text{ Sa}}/{\text{b}}$$

where b denotes the slope of the calibration plot. The obtained values for LOD and LOQ demonstrated the sensitivity of the proposed approaches (Table 1).

#### Accuracy and precision

Both BUP and MLX raw materials were analyzed using the suggested procedures over the specified concentration ranges. The excellent accuracy of the suggested procedures was confirmed by the high values of % recoveries and the low values of % Error (Table 2). Moreover, by measuring three distinct drug concentrations at three different time intervals throughout the same day and over the course of three different days, respectively, the intra-day and inter-day precisions were confirmed. Low percentages of RSD and error were evidence of the proposed methods’ precision, as displayed in Table 3.

**Table 2:** Results of the application of the developed approaches for the estimation of BUP and MLX raw materials.

Parameters	Drug	BUP			MLX		
	Method	D^2^	RS	λ_max_		λ_max_	UV direct
		at (245 nm)	at (224.7 nm)	at (224.7 nm)		at (224.7 nm)	at (359.3 nm)
	Taken Conc. (µg/mL)	Found^a^ Conc. (µg/mL)	Found^a^ Conc. (µg/mL)	Found^a^ Conc. (µg/mL)	Taken Conc. (µg/mL)	Found^a^ Conc. (µg/mL)	Found^a^ Conc. (µg/mL)
	5.0	4.973	5.000	4.943	1.0	0.995	1.016
	10.0	9.974	9.901	9.887	2.0	1.969	2.015
	20.0	19.975	20.377	20.085	4.0	4.09	4.058
	30.0	29.728	30.415	30.094	6.0	5.996	6.045
	50.0	50.714	50.25	79.811	8.0	8.03	7.893
	60.0	60.221	59.335	50.103	10.0	9.808	9.902
	80.0	79.73	80.999	59.335	15.0	15.064	15.09
Mean (X¯)		99.95	100.42	99.62		99.86	100.40
± SD		0.76	1.18	0.73		1.39	1.14
% RSD		0.762	1.170	0.730		1.392	1.132
% Error		0.288	0.444	0.275		0.525	0.430

^a^Each result is the average of three separate determinations.

**Table 3:** Results of precision study for the estimation of BUP and MLX by the developed approaches.

Drugs	Drug conc. µg/mL	Method	Intra-day				Inter-day			
			% Found^a^	± SD	% RSD	% Error	% Found^a^	± SD	% RSD	% Error
BUP	20.0	D^2^	100.13	0.513	0.512	0.296	99.73	0.493	0.495	0.286
	30.0		100.15	0.673	0.672	0.388	99.8	0.492	0.493	0.285
	50.0		99.83	0.808	0.81	0.468	99.81	0.415	0.416	0.24
	20.0	RS	100.2	0.721	0.72	0.416	100.23	0.208	0.208	0.12
	30.0		99.9	0.625	0.625	0.361	99.87	0.503	0.504	0.291
	50.0		99.63	0.404	0.406	0.234	99.77	0.351	0.352	0.203
MLX	4.0	UV direct	99.87	0.681	0.682	0.394	100.2	0.656	0.654	0.378
	6.0		100.17	0.321	0.321	0.185	99.7	0.436	0.437	0.252
	8.0		99.33	0.777	0.782	0.451	99.54	0.627	0.63	0.364

^a^Each result is the mean of 3 separate determinations.

#### Selectivity

The determination of BUP and MLX in their prepared co-formulated ampoules was achieved using the suggested procedures. The developed spectrophotometric methods were successfully employed to determine BUP and MLX without any interference from the excipients. The outcomes demonstrate the selectivity of the suggested procedures as revealed by the high % recoveries and the low % RSD values shown in Table 4.

**Table 4:** Analysis outcomes for the estimation of BUP and MLX in prepared ampoules using the developed approaches.

Mix. No	Taken conc. (µg/mL)	D^2^	RD	AF	UV-direct	
		Found^a^ conc. (µg/mL)	Found^a^ conc. (µg/mL)	Found^a^ conc. (µg/mL)	Taken Conc. (µg/mL)	Found^a^ Conc. (µg/mL)
					BUP	MLX
1	33.3	33.1	33.03	33.1	1.0	0.99
2	50.0	50.1	50.25	50.4	1.5	1.51
3	66.6	66.27	66.4	66.87	2.0	2.00
Mean		99.7	99.8	100.2		99.97
± S.D		0.436	0.656	0.721		0.666
% RSD		0.437	0.657	0.72		0.666
% Error		0.252	0.379	0.416		0.385

^a^Each result is the average of three separate determinations.

### Methods applications

#### Assay of ampoules

The estimation of various ratios of BUP and MLX in their prepared ampoules was successfully accomplished using the suggested spectrophotometric techniques. The obtained high percent recoveries and low percent RSD values show the high selectivity of the developed methods (Table 4). Therefore, they can be better suited for the routine analysis of the cited drugs in quality control laboratories.

### Evaluation of greenness of the proposed methods

**Fig. 6:**
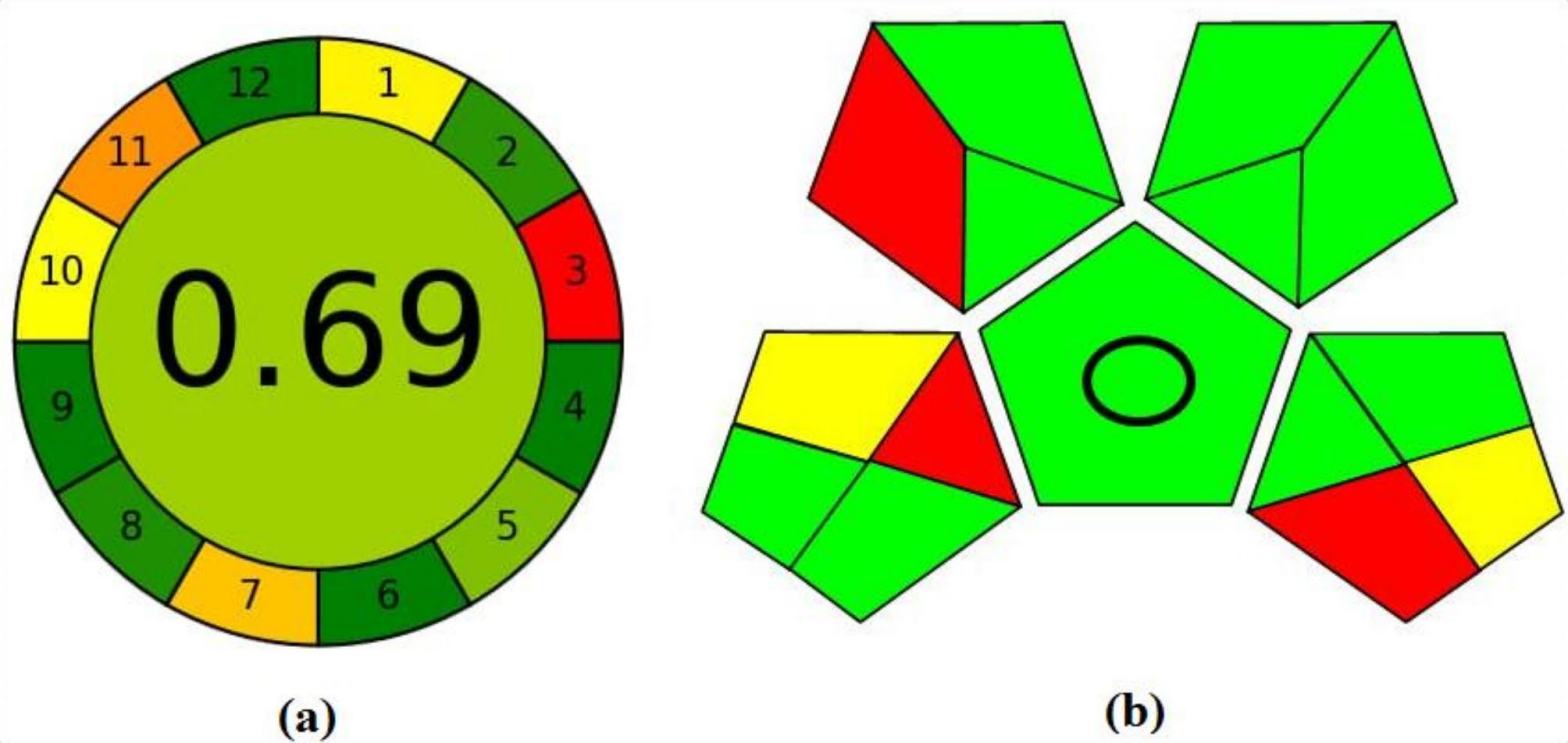
Evaluation of the greenness profile of the proposed methods using (**a**) AGREE, (**b**) GAPI.

Analysts have an essential role in both human and environment protection from solvents and organic hazardous produced from pharmaceutical and chemical preparations. Therefore, different greenness evaluation metrics were developed to assess the green profiles of the analytical methods^28–38^. From these methods, two tools were applied to evaluate the proposed methods’ greenness, including AGREE and GAPI^25,26^. By weighing 12 important criteria, AGREE is a tool for identifying the occupational and environmental hazards included in the analytical process. The range of the result is 0 to 1.0. Figure 6a illustrates how the approaches generated positive results and a high score (0.69), showcasing adequate green methodologies. Additionally, GAPI was used, and as presented in Fig. 6b, the developed methods are direct methods (no extraction procedure). Small quantities of non-toxic chemicals with minimal waste are required for all techniques. The developed methods also include qualification and quantification.

## Conclusion

In order to simultaneously analyze BUP and MLX in a challenging ratio of 33.3:1.0, four straightforward, accurate, and green spectrophotometric methods were developed. These methods included D^2^, RD, RS, and AF (for BUP), as well as direct UV approaches (for MLX). The developed procedures proved to be suitable for the routine analysis of BUP and MLX in their co-formulated ampoules with high % recoveries and low % RSD values. The proposed univariate approaches offer wider linearity ranges for both medications and call for less in the way of mathematical manipulation, which gives the suggested approaches various merits over the reported chromatographic ones. In addition, the developed spectrophotometric approaches are more affordable and straightforward, requiring less expensive instruments, don’t need prior separation steps or high volumes of organic solvents, and are more environmentally friendly than the previously reported techniques used for concurrent assay of BUP and MLX.

## Data Availability

The datasets generated and/or analyzed during the current study are available from the corresponding author on reasonable request.
